# Can essential oils effectively control skin bacteria? Unveiling their powerful antimicrobial effects

**DOI:** 10.1128/spectrum.01723-25

**Published:** 2025-11-25

**Authors:** Ana I. Lopes, Cláudia S. Oliveira, Manuela E. Pintado, Freni K. Tavaria

**Affiliations:** 1 Universidade Católica Portuguesa, CBQF - Centro de Biotecnologia e Química Fina – Laboratório Associado, Escola Superior de Biotecnologia 112064, Porto, Portugal; City of Hope Department of Pathology, Duarte, California, USA

**Keywords:** essential oils, skin bacteria, *Staphylococcus* spp, *Cutibacterium acnes*, antibacterial activity

## Abstract

Skin-associated bacteria play a significant role in various dermatological conditions. Essential oils (EOs), known for their broad-spectrum antimicrobial properties, have emerged as promising alternatives to conventional treatments for bacterial skin infections. However, studies on the potential of EOs for treating these infections have largely centered on *Staphylococcus aureus*. As such, this study evaluated the antimicrobial activity of six EOs—rosemary, eucalyptus, lavender, basil, sage, and thyme—against methicillin-sensitive *S. aureus* (MSSA), methicillin-resistant *S. aureus* (MRSA), *Staphylococcus epidermidis*, and *Cutibacterium acnes*. Among the tested EOs, thyme EO exhibited the strongest antibacterial activity with the lowest minimum inhibitory concentration (MIC; 0.07–2.81 mg/mL) and (minimum bactericidal concentration (MBC; 0.07–11.22 mg/mL) values. In contrast, basil EO displayed the weakest activity, with the smallest inhibition zones observed against *S. aureus* MSSA (37.00 ± 1.80 mm), MRSA (34.50 ± 10.40 mm), and *S. epidermidis* (35.30 ± 1.30 mm), as well as the highest MIC (12.11–24.22 mg/mL) and MBC (12.11 to >24.22 mg/mL) values. Eucalyptus and lavender EOs showed MIC values ranging from 5.67 to 22.65 mg/mL and 5.54 to 22.16 mg/mL, respectively, and corresponding MBC values from 5.67 to >22.65 mg/mL and 5.54 to >22.16 mg/mL, though their activity was lower than that of thyme EO. Further analysis using flow cytometry confirmed the antibacterial activity of these oils, revealing that their mechanism of action involves disrupting bacterial membrane integrity. *S. epidermidis* exhibited the greatest resistance to EO treatments, and *C. acnes* was the most susceptible species. Additionally, the ability of EOs to inhibit MRSA highlights their potential as an alternative treatment for drug-resistant skin infections.

**IMPORTANCE:**

Essential oils (EOs) are widely known and often used to support pain relief, better sleep, immune health, and protection against germs or inflammation. In this study, we tested six EOs to see how effectively they could fight bacteria linked to common skin issues like acne, dermatitis, and eczema. Thyme oil showed the strongest antibacterial effect, while basil oil was the weakest. These oils work by damaging bacterial membranes, leading to bacterial death. Their effectiveness depends on the type of bacteria, so they could be used alone or combined with standard treatments. Eucalyptus and lavender oils also performed well, suggesting they may complement existing therapies for skin infections.

## INTRODUCTION

Skin is the largest organ of the human body, covering approximately 1.8 m^2^ and accounting for about 15% of total body mass (1, 2). Along with its appendages—nails, hairs, and glands—it serves as a crucial physical and immunologic barrier that protects the body against pathogenic microorganisms and harmful UV radiation. Furthermore, the skin regulates water loss through evaporation, plays a key role in thermoregulation, and facilitates sensory perception, contributing to overall skin homeostasis (3–5).

The skin microbiota, composed of millions of bacteria, fungi, viruses, and mites (6), plays a crucial role in maintaining skin barrier function through complex microbe-microbe and host-microbe interactions (4). Among the key bacterial species are *Staphylococcus* species and *Cutibacterium acnes*, both of which contribute to skin homeostasis.

*Staphylococcus* spp. is one of the most abundant bacterial genera of the human skin microbiome (4). These Gram-positive, facultatively anaerobic, and non-motile bacteria typically form clusters and exhibit remarkable adaptability, thriving across diverse temperatures and salt concentrations (7–9). The genus includes both opportunistic pathogens, such as *Staphylococcus aureus*, and commensal species, such as *Staphylococcus epidermidis* (10).

*S. aureus* is one of the major opportunistic pathogens known for its ability to develop resistance mechanisms and produce various virulence factors. This bacterium is commonly associated with superficial wound infections (11, 12), with methicillin-resistant *S. aureus* (MRSA) strains being the primary cause of infections in community settings. Infections by MRSA strains pose a greater clinical and economic burden due to higher treatment costs and limited therapeutic options (11).

In contrast, *S. epidermidis* is a commensal member of the skin microbiota, predominantly residing on the epidermal basement membrane across dry, moist, and sebaceous areas (13, 14). It plays a protective role by producing antimicrobial molecules, namely modulins and lipopeptides, which prevent colonization by pathogenic microbes (10).

Similarly, *C. acnes* is an anaerobic, Gram-positive bacterium that primarily inhabits sebaceous glands. It contributes to skin homeostasis by breaking down triglycerides into fatty acids, which help regulate skin pH and inhibit the growth of harmful microorganisms (5).

Both *S. epidermidis* and *C. acnes* are typically considered commensal bacteria that support the maintenance of a healthy skin barrier. They play essential roles in preventing microbiota imbalances, protecting against pathogen invasion, and contributing to skin homeostasis through the production of beneficial metabolites. These bacteria share a symbiotic and mutualistic relationship with the skin, benefiting both the resident microorganisms and the host (14). However, when the equilibrium between commensal and pathogenic microorganisms is disrupted—a condition known as dysbiosis—these otherwise beneficial bacteria can become pathogenic. In such cases, microbial overgrowth or altered interactions may contribute to various skin diseases, such as atopic dermatitis, eczema, acne, and more (10).

Currently, treatments for these conditions involve the use of a set of therapies that include emollients, anti-inflammatory drugs (such as corticosteroids), and antimicrobial agents (15). However, many of these drugs eventually fail. Antimicrobial agents are often associated with the acquisition of resistance due to their extensive and continued use (16), while anti-inflammatory drugs may lead to side effects such as skin thinning, irritation, and suppression of the skin’s natural immune response (10). As such, researchers have been exploring more natural, alternative options, such as essential oils (EOs).

EOs are plant-derived substances rich in bioactive compounds, renowned for their antimicrobial, anti-inflammatory, and healing properties (17). Due to their complex chemical composition, EOs’ antimicrobial activity is multifaceted, targeting bacterial cell membranes and interfering with essential cellular processes, which reduces the likelihood of resistance development (18), allowing EOs to effectively target pathogens while preserving the skin’s beneficial microbes. EOs disrupt membrane integrity, causing leakage of ATP and metabolites, enzyme dysfunction, cytoplasmic coagulation, and damage to genetic material (17). They may also interfere with quorum sensing, thereby reducing biofilm formation, proteolytic activity, and swarming motility (17). Beyond these antimicrobial effects, EOs also enhance skin regeneration and alleviate inflammation, making them a versatile and promising solution for managing a range of both acute and chronic skin conditions (19).

Research into the potential of EOs for treating bacterial skin infections has predominantly focused on *S. aureus*, with significant attention given to its antimicrobial susceptibility and mechanisms of action. In contrast, other skin-associated bacteria, such as *S. epidermidis* and *C. acnes*, have been comparatively underexplored (10). To address this gap, this study evaluated the antimicrobial activity of six EOs—rosemary, eucalyptus, lavender, basil, sage, and thyme—against four bacteria commonly found in the skin microbiota: methicillin-sensitive *S. aureus* (MSSA), MRSA, *S. epidermidis*, and *C. acnes*.

## MATERIALS AND METHODS

### Materials

Culture media: Müller-Hinton (MH) broth and agar culture media were acquired from BIOKAR Diagnostics (Allonne, France), and anaerobe basal broth was obtained from Oxoid (Hampshire, United Kingdom).

Antimicrobial agents: Rosemary, eucalyptus, lavender, basil, sage, and thyme EOs were purchased from Socidestilda Lda. (Setúbal, Portugal). The chemical composition of each EO was retrieved from the supplier’s data associated with the respective CAS numbers and is presented in Table 1. Chloramphenicol (30 µg), gentamicin (120 µg), and vancomycin (30 µg) antibiotic discs were purchased from BioMérieux (Marcy-l'Étoile, France), and chloramphenicol and erythromycin powders were acquired from Sigma-Aldrich (St. Louis, MO, USA).

**TABLE 1:** Chemical composition of the EOs used in the study

Chemical composition of EOs					
Rosemary (CAS 84604-14-8)	Eucalyptus (CAS 84625-32-1)	Lavender (CAS 91722-69-9)	Basil (CAS 84775-71-3)	Sage (CAS 84776-73-8)	Thyme (CAS 85085-75-2)
Cineole (eucalyptol)	Cineole (eucalyptol)	2-isopropenyl-5-methylhex-4-enyl acetate	(R)−3,7-dimethyl-1,6-octadien-3-ol	α-Thujene	Thymol
Pin-2(3)-ene	Dipentene	(1S,4S,4aR,8aR)−1,6-dimethyl-4-propan-2-yl-3,4,4a,7,8,8a-hexahydro-2H-naphthalen-1-ol	Cineole (eucalyptol)	α-Pinene	p-Cymene
Bornan-2-one	Pin-2(3)-ene	Caryophyllene	2,6-Dimethyl-6-(4-methyl-3-pentenyl)bicyclo[3.1.1]hept-2-ene	Camphene	p-Mentha-1,4-diene
Dipentene	p-Cymene	(R*,R*)-α,4-dimethyl-α-(4-methyl-3-pentenyl)cyclohex-3-ene-1-methanol	Eugenol	Sabinene	Linalool
Terpineol	p-Mentha-1,4-diene	Cineole (eucalyptol)	(1Z,6Z)−1-methyl-5-methylidene-8-(propan-2-yl)cyclodeca-1,6-diene	β-Pinene	Carvacrol
p-Menth-1-en-8-ol	p-Mentha-1,5-diene	p-Menth-1-en-4-ol	7-Methyl-4-methylidene-1-propan-2-yl-2,3,4a,5,6,8a-hexahydro-1H-naphthalene	β-Thujene	7-Methyl-3-methyleneocta-1,6-diene
Pin-2(10)-ene	7-Methyl-3-methyleneocta-1,6-diene	(1S-endo)−1,7,7-trimethylbicyclo[2.2.1]heptan-2-ol	(1R,4S,4aR)−1,6-dimethyl-4-propan-2-yl-3,4,4a,7,8,8a-hexahydro-2H-naphthalen-1-ol	Viridiflorol	p-Mentha-1,3-diene
p-Mentha-1,4-diene	Pin-2(10)-ene	7-Methoxycoumarin	(1α,2β,4β)−1-Methyl-2,4-bis(methylvinyl)−1-vinylcyclohexane	Humulene epoxide II	Caryophyllene
p-Cymene	p-Menth-1-en-8-ol	(+)-Bornan-2-one	(3S,3aS,5R)−3,8-dimethyl-5-prop-1-en-2-yl-1,2,3,3a,4,5,6,7-octahydroazulene	Carylophylla-4(12),8(13)-dien-5α-ol	Pin-2(3)-ene
p-Mentha-1,5-diene		Coumarin	4-Allylanisole	Manool	5-Isopropyl-2-methylbicyclo[3.1.0]hex-2-ene
7-Methyl-3-methyleneocta-1,6-diene		(R)−3,7-Dimethyl-1,6-octadien-3-ol	7-Methyl-3-methyleneocta-1,6-diene		p-Menth-1-en-4-ol
Camphene		Linalyl acetate	1-Methyl-4-(1-methylethylidene)−2-(1-methylvinyl)−1-vinylcyclohexane		DL-Borneol
Caryophyllene					5-Isopropyl-2-methylanisole
4,6,6-Trimethylbicyclo[3.1.1]hept-3-en-2-one					
(1S-endo)−1,7,7-trimethylbicyclo[2.2.1]heptan-2-ol					
Linalool					
p-Menth-1-en-4-ol					
L-Born-2-yl acetate					

Reagents: Iodonitrotetrazolium chloride (INT) and phosphate-buffered saline (PBS) were purchased from Sigma-Aldrich (St. Louis, MO, USA). Methanol (99% [vol/vol]), glacial acetic acid, and crystal violet were acquired from Merck (Darmstadt, Germany). The BD Cell Viability Kit for flow cytometry (consisting of thiazole orange [TO; 420 nmolL^−1^] and propidium iodide [PI; 43 μmolL^−1^]) was acquired from BD Biosciences (Franklin Lakes, NJ, USA).

### Microorganisms

The microorganisms used in this study were MSSA (ATCC 6538), MRSA (DSM 11729), *S. epidermidis* (DSM 20044), and *C. acnes* subsp. *acnes* (DSM 1897). These bacterial species were selected to represent both commensal and pathogenic members of the human skin microbiota (10, 14). The microbial cultures were maintained in MH broth for *Staphylococcus* species or anaerobic basal broth for *C. acnes* until antimicrobial activity testing.

### Disc diffusion assay

The disc diffusion assay was conducted following the Kirby–Bauer agar disk diffusion method, as reported by the Clinical and Laboratory Standards Institute (CLSI) (20), with some modifications.

*Staphylococcus* spp.: For *Staphylococcus* species, MH agar plates with a 4.0 ± 0.5 mm depth were prepared and allowed to solidify. An inoculum in MH broth containing approximately 10^8^ colony-forming units (CFU) mL^−1^ was prepared and uniformly spread over the agar surface using a sterile cotton swab. After that, filter paper disks (Whatman no. 2) with a diameter of 6 mm were placed on the top of the agar surface, and 15 µL of each EO was applied to the discs. The plates were refrigerated at 4°C for 15 min to allow diffusion and incubated at 37°C for 24 h in aerobiosis. Following incubation, the zones of inhibition, represented by a clear halo around the disks, were measured.

*C. acnes*: For *C. acnes*, the test was performed by incorporating the bacterial inoculum directly into the agar medium. The bacterium was first incubated at 37°C under anaerobic conditions for 72 h in anaerobe basal broth. Anaerobe basal agar was then prepared, sterilized, and cooled to approximately 45°C. After that, the inoculum was incorporated into the agar at a final concentration of 10% (vol/vol) and poured into plates. After the medium solidified, paper discs were placed on the agar surface, and 15 µL of each EO was applied to the discs. The plates were refrigerated at 4°C for 15 min and incubated at 37°C under anaerobic conditions for 72 h. After incubation, the resulting zones of inhibition were measured.

For both *Staphylococcus* spp. and *C. acnes*, chloramphenicol (30 µg), gentamicin (120 µg), and vancomycin (30 µg) antibiotic discs were included as positive controls. Controls consisted of plates with the inoculated microorganism but without any antimicrobial agent.

All antimicrobial substances were tested in duplicate, and two independent tests were performed.

### Determination of minimum inhibitory and minimum bactericidal concentrations

The minimum inhibitory concentrations (MICs) of the EOs were determined using the microdilution method combined with a rapid INT colorimetric assay, as described by Alves et al. (21), with some modifications.

Initially, serial dilutions of each EO, ranging from 5% to 0.078% (vol/vol), were prepared in MH broth for *Staphylococcus* spp. and anaerobe basal broth for *C. acnes*. To ensure homogeneity, the EOs were mixed directly into the culture media and vortexed vigorously immediately before inoculation. Subsequently, 50 µL of each dilution was dispensed into 0.2 mL tubes, followed by inoculation with 50 µL of a bacterial suspension standardized to 10^6^ CFU mL^−1^. A positive control containing 50 µL of bacterial inoculum and 50 µL of the appropriate medium was included; a negative control with 100 µL of medium (no bacteria) was prepared.

All tubes containing *Staphylococcus* spp. were incubated at 37°C for 24 h in aerobiosis, while tubes containing *C. acnes* were incubated at 37°C for 72 h under anaerobic conditions.

After incubation, 40 µL of an INT solution (0.2 mg/mL) was added to each tube and incubated at 37°C for 30 min. The INT dye transitions from yellow to pink in the presence of viable bacterial cells, allowing MIC to be defined as the lowest EO concentration that prevents a color change. All EO dilutions and controls were tested in triplicate, and three independent tests were performed. MIC values were expressed in mg/mL and calculated based on the density of each oil.

To determine the minimum bactericidal concentrations (MBCs), 50 µL from each tube showing no color change was plated onto MH agar (*Staphylococcus* spp.) or anaerobe basal agar plates (*C. acnes*). The plates were incubated at 37°C for 24 h in aerobiosis for *Staphylococcus* spp. and 37°C for 72 h under anaerobic conditions for *C. acnes*. The MBC was defined as the lowest EO concentration that completely inhibited bacterial growth. Each concentration was tested in triplicate, and three independent tests were conducted.

The MIC and MBC values of chloramphenicol and erythromycin antibiotics were also determined using the same methodology and compared with the results obtained for the EOs.

### Inhibition curves

The growth inhibition curves for each EO were determined at three concentrations, 2× MIC, MIC, and ½× MIC, following the methodology described by Alexandre et al. (22).

For *Staphylococcus* species, EOs were diluted to the desired concentrations in MH broth. Then, 50 µL of each dilution was pipetted into a 96-well microplate (Sarstedt, Numbrecht, Germany) and inoculated with 50 µL of an overnight bacterial liquid inoculum at 10^8^ CFU mL^−1^. Optical density (OD) at 600 nm was measured hourly over 24 h at 37°C in aerobiosis using a microplate reader (Multiskan GO, Thermo Scientific, Vantaa, Finland). Aliquots of the bacterial inoculum in MH broth served as positive controls, while aliquots of the media without inoculum acted as negative controls.

For *C. acnes*, each EO was diluted in anaerobe basal broth. Then, 75 µL of each dilution was pipetted into a 96-well microplate and inoculated with 75 µL of a 72 h growth inoculum at 10^8^ CFU mL^−1^. To maintain anaerobic conditions, 50 µL of liquid paraffin was pipetted on the top of each well. Then, using the same microplate reader, the OD at 600 nm was measured for 72 h (3-h intervals) at 37°C. Positive controls included bacterial inoculum in anaerobe basal broth, whereas negative controls contained only the medium without bacteria.

A higher inoculum density, approximately 100 times greater than that used for MIC and MBC determinations, was intentionally employed in these assays to ensure measurable and reproducible growth kinetics suitable for calculating inhibition percentages under dynamic conditions.

Bacterial growth was inferred from increases in OD values. Each condition was tested in triplicate to ensure reproducibility.

The inhibition percentages for each EO at all tested concentrations were calculated after 24 h for *Staphylococcus* spp. and 72 h for *C. acnes*, using equation (1):


(1) $$Inhibition\;percentage\;\left(\%\right)=\left(\frac{OD_{C}-OD_{EO}}{OD_{C}}\right)\times 100$$

where OD_C_ represents the bacterial culture’s OD in the positive control at the end of the assay, and OD_EO_ represents the OD of the bacterial culture exposed to the EO.

This calculation provides the percentage reduction in bacterial growth relative to the untreated control, enabling a quantitative assessment of the antimicrobial efficacy of each EO.

### Antibiofilm formation activity

The ability of EOs to inhibit biofilm formation was evaluated at three concentrations—MIC, ½× MIC, and ¼× MIC—using a modified version of the methodology described by Costa et al. (23).

First, EOs were diluted to the desired concentrations in MH broth (*Staphylococcus* spp.) and anaerobe basal broth (*C. acnes*). Then, 100 µL of each dilution was pipetted into a 96-well flat-bottom microplate (Nunc, Darmstadt, Germany) and inoculated with 100 µL of an overnight bacterial liquid inoculum at 10^8^ CFU mL^−1^ (*Staphylococcus* spp.) or a 72 h growth inoculum at 10^8^ CFU mL^−1^ (*C. acnes*). A higher bacterial inoculum was employed in this assay than in the MIC/MBC tests to promote consistent and reproducible biofilm formation. This inoculum density ensures uniform cell adherence and matrix development, enabling a reliable assessment of the EOs’ capacity to inhibit biofilm establishment. Aliquots of the bacterial inoculum in the appropriate broth media were used as positive controls, and aliquots of the media without inoculum were used as negative controls to verify sterility. The plates were incubated at 37°C for 16–20 h in aerobiosis for *Staphylococcus* species and 37°C for 60–64 h under anaerobic conditions for *C. acnes*.

After incubation, non-adherent cells were removed by discarding the contents of the wells and washing them gently with deionized water. The adhered biofilm-forming cells were fixed with 200 µL of methanol for 15 min, followed by methanol removal and air-drying of the wells. Biofilms were stained with 0.1% (vol/vol) crystal violet solution, and excess stain was removed by rinsing under tap water, followed by air-drying.

To quantify biofilm biomass, the crystal violet stain was solubilized with 33% (vol/vol) acetic acid for 15 min under constant agitation. The OD of the wells was measured at 595 nm using a Synergy H1 plate reader (Vermont, USA). The percentage inhibition of biofilm formation was calculated using the following equation (2):


(2) $$\%\;biofilm\;formation\;inhibition\;=100-\;\left(\frac{ODassay}{ODControl}\right)\times 100$$

Each condition was tested in quadruplicate, and three independent tests were performed.

### Assessment of membrane damage using flow cytometry

The damage in the cell membrane was assessed by flow cytometry using eucalyptus, lavender, and thyme EOs, as these oils demonstrated the best results in previous assays. Three concentrations—MIC, ½× MIC, and ¼× MIC—were analyzed for each oil. The experimental procedure involved the following steps:

Exposure of the bacterial cells to EOs: EOs were diluted to the desired concentrations in MH broth (*Staphylococcus* spp.) and anaerobe basal broth (*C. acnes*). Then, 250 µL of each dilution was pipetted into 1.5 mL tubes and inoculated with 250 µL of an overnight bacterial liquid inoculum at 10^8^ CFU mL^−1^ (*Staphylococcus* spp.) or a 72-h growth inoculum at 10^8^ CFU mL^−1^ (*C. acnes*). A bacterial growth control was also prepared by inoculating 250 µL of culture medium with 250 µL of the inoculum. All tubes were incubated at 37°C in aerobiosis for 14 h (*Staphylococcus* spp.) or 37°C under anaerobic conditions for 48 h (*C. acnes*), corresponding to the end of the exponential growth phase.

Staining of Bacterial Cells for Flow Cytometry: The bacterial cells were treated following the procedure of Melo et al. (24) with some modifications. After incubation, the cells were harvested by centrifugation (5,000 rpm; 10 min), and the pellet was washed with PBS and centrifuged again (5,000 rpm; 10 min). Then, the cells were resuspended in 500 µL of PBS, stained with 5 µL of TO and 5 µL of PI, and incubated at room temperature in the dark for at least 5 min, following the manufacturer’s instructions (BD Cell Viability Kit, BD Biosciences, USA). The stained samples were analyzed immediately using a BD Accuri C6 flow cytometer (BD Biosciences, Franklin Lakes, NJ, USA). A dot plot with FL1 (to detect TO) and FL3 (to detect PI) was used to differentiate live, injured, and dead bacterial populations, following the recommendations of the BD Cell Viability Kit. Three independent tests were carried out for each condition.

### Statistical analysis

The results were presented as mean ± standard deviation (SD). Statistical analysis was conducted using GraphPad Prism 8.0 (GraphPad Software, San Diego, California, USA). A one-way analysis of variance was performed, followed by Tukey’s HSD post hoc test, after confirming homoscedasticity.

## RESULTS AND DISCUSSION

### Initial antimicrobial screening of EOs

The increasing prevalence of antibiotic resistance has intensified the search for new antimicrobial agents, including EOs (25). As a result, the scientific community has increasingly focused on efficient screening methods to identify effective alternative conventional treatments. Among these methods, the disc diffusion assay is widely used due to its cost-effectiveness, simplicity, and adaptability for testing a broad range of microorganisms (12).

In this study, the disc diffusion assay was employed as an initial screening tool to evaluate the antimicrobial potential of six EOs—rosemary, eucalyptus, lavender, basil, sage, and thyme EOs against four selected skin-associated bacteria, including MSSA, MRSA*, S. epidermidis,* and *C. acnes* (Table 2). In addition, to establish a comparative baseline, the same assay was performed using selected conventional antibiotics, providing insight into the relative efficacy of EOs against these clinically relevant bacteria (Table 2).

**TABLE 2:** Inhibition halos (mm) of EOs for skin bacteria and susceptibility of skin bacteria to antibiotics determined by disc diffusion (mm)*^a^*^,^*^b^*^,^*^c^*

Inhibition halos (mm)				
EOs	MSSA	MRSA	*S. epidermidis*	*C. acnes*
Rosemary	44.50 ± 6.90^a^	43.80 ± 8.50^a^	47.00 ± 11.5^a^	19.80 ± 4.10^a^
Eucalyptus	47.80 ± 5.60^a^	35.00 ± 4.10^a,c^	50.50 ± 4.90^a,b^	30.00 ± 4.70^b^
Lavender	TI	42.50 ± 6.10^a,c^	51.30 ± 4.80^a^	23.80 ± 10.3^a^
Basil	37.00 ± 1.80^a,b^	34.50 ± 10.4^a^	35.30 ± 1.30^a,c^	21.50 ± 1.70^a^
Sage	53.30 ± 7.40^a,b^	65.00 ± 9.10^b^	47.30 ± 2.50^a^	32.50 ± 2.90^b^
Thyme	TI	TI	TI	TI

Antibiotics	MSSA	MRSA	*S. epidermidis*	*C. acnes*
Chloramphenicol	S (32.00 ± 2.45)	R (≤12.00)	S (31.50 ± 2.12)	S (23.5 ± 0.50)
Gentamicin	S (31.00 ± 2.00)	S (31.25 ± 2.63)	S (33.75 ± 0.96)	I (15.5 ± 0.50)
Vancomycin	S (19.25 ± 0.96)	S (18.75 ± 1.50)	S (19.75 ± 1.71)	S (23.75 ± 3.56)

^*a*^ Means ± standard deviation within the same column labeled with the same letter do not statistically differ from each other (*P* > 0.05).

^*b*^ TI, total inhibition, means that there is a clear zone (halo) with no growth at the tested volume (15 µL).

^*c*^ According to CLSI guidelines (20), the interpretive criteria for *Staphylococcus* species are as follows: Chloramphenicol (30 µg): Susceptible (S): ≥18 mm; Intermediate (I): 13–17 mm; Resistant (R): ≤12 mm; Gentamicin (120 µg): Susceptible (S): ≥16 mm; Intermediate (I): 13–15 mm; Resistant (R): ≤12 mm; Vancomycin (30 µg): Susceptible (S): ≥15 mm; Intermediate (I): 14 mm; Resistant (R): ≤13 mm. For *C. acnes*, standardized interpretive criteria are not well established in CLSI guidelines. As such, the results were interpreted using the same criteria of *Staphylococcus* spp.

The results revealed that all tested EOs exhibited antimicrobial activity, producing inhibition zones comparable to or, in some cases, larger than those of standard antibiotics. Among the tested bacteria, thyme demonstrated the most potent antibacterial effect, completely inhibiting all strains. For MSSA, inhibition zones ranged from 37.00 ± 1.80 mm (basil) to 53.30 ± 7.40 mm (sage), with lavender EO achieving complete inhibition. For MRSA*,* the inhibition halos varied between 34.50 ± 10.40 mm (basil) and 65.00 ± 9.10 mm (sage). For *S. epidermidis*, basil exhibited the smallest inhibition zone of 35.30 ± 1.30 mm, while lavender EO displayed the largest (51.30 ± 4.80 mm). In the case of *C. acnes*, inhibition zones ranged from 19.80 ± 4.10 mm (rosemary) to 32.50 ± 2.90 mm (sage) (Table 2). These findings highlight the strong antimicrobial potential of EOs, particularly thyme EO, which exhibited broad-spectrum activity against all tested bacteria (Table 2).

Several studies have demonstrated the antibacterial potential of EOs against skin-associated bacteria, especially *S. aureus*. For instance, Gheorghita et al. (26) reported inhibition zones of 6 mm for eucalyptus, 12 mm for sage, and 30 mm for thyme against a strain of MSSA. In the present study, these EOs also exhibited antimicrobial activity, with inhibition halos that were even larger than those previously reported. Notably, Gheorghita et al. (26) also found that thyme EO produced a larger inhibition zone than chloramphenicol, a result that aligns with the findings of this study. The potent antibacterial activity of thyme EO against MSSA has been further corroborated by Ebani et al. (27) and Brożyna et al. (28), reinforcing its potential as an effective natural antimicrobial agent.

Brożyna et al. (28) also assessed the antibacterial activity of rosemary, eucalyptus, lavender, and basil EOs, reporting inhibition zones ranging from 10 mm (lavender) to 30 mm (eucalyptus). These values were lower than those obtained in the present study. The same authors (28) further evaluated the antimicrobial effects of rosemary, eucalyptus, lavender, basil, and thyme EOs against MRSA, with inhibition zones ranging from 3 mm to 43 mm. Thyme exhibited the largest inhibition area, a finding consistent with the results of this study. Notably, for the remaining EOs, the inhibition halos observed in this study were larger than those reported by Brożyna et al. (28).

To the authors’ knowledge, studies utilizing the disc diffusion method to assess the antimicrobial activity of EOs against *S. epidermidis* and *C. acnes* remain scarce. Esmael et al. (29) investigated the antimicrobial activity of rosemary, lavender, and thyme oils against *S. aureus*, *S. epidermidis,* and *C. acnes*, reporting inhibition halos of 12.5 mm for *S. aureus*, 15.18 mm for *S. epidermidis*, and 14.77 mm for *C. acnes* with rosemary EO. These values were lower than those obtained in this study. Moreover, Esmael et al. (29) found that all tested bacteria were resistant to lavender and thyme EOs, a result that contrasts with the present findings. In this study, no bacterial strain exhibited resistance to any EOs, and thyme EO completely inhibited all tested bacterial species, and lavender EO fully inhibited MSSA.

EOs, as natural products, inherently exhibit significant variability in their composition, concentration, and overall quality. This variability is influenced by multiple factors, including climate conditions, soil composition, the specific plant part used for extraction, the plant’s age, and the stage of its vegetative cycle (30). As a result, even when the same EO is tested against identical bacterial strains under similar experimental conditions, differences in antimicrobial activity may arise. This variability likely explains the discrepancies observed across different studies, highlighting the importance of standardizing EO extraction and characterization methods to ensure reproducibility and consistency in research findings.

### Determination of MIC and MBC

While the disc diffusion assay is a widely used method for evaluating the antimicrobial potential of EOs, it remains a qualitative technique that does not quantify the exact concentration of antimicrobials diffusing into the agar medium (12). To address this limitation, the broth microdilution assay was employed to determine the MIC of each EO—the lowest concentration required to inhibit visible bacterial growth. Additionally, the MBC, which represents the lowest concentration needed to kill the bacteria, was assessed. The results of these analyses are summarized in Table 3. For comparative purposes, the same test was performed on selected conventional antibiotics, as presented in Table 3. This approach provides a more precise evaluation of EO efficacy, enabling a direct comparison with standard antimicrobial agents.

**TABLE 3:** MIC and MBC values (mg/mL) of EOs and antibiotics for skin bacteria

	MSSA		MRSA		*S. epidermidis*		*C. acnes*	
EOs	MIC	MBC	MIC	MBC	MIC	MBC	MIC	MBC
Rosemary	22.53	22.53	22.53	22.53	22.53	>22.53	5.63	5.63
Eucalyptus	22.65	>22.65	22.65	>22.65	22.65	>22.65	5.67	5.67
Lavender	22.16	>22.16	22.16	>22.16	22.16	>22.16	5.54	5.54
Basil	12.11	24.22	12.11	>24.22	24.22	>24.22	12.11	12.11
Sage	22.66	22.66	22.66	>22.66	22.66	>22.66	22.66	22.66
Thyme	2.81	5.61	0.70	0.70	2.81	11.22	0.70	0.70

Antibiotics	MIC	MBC	MIC	MBC	MIC	MBC	MIC	MBC
Chloramphenicol	0.016	0.016	0.125	0.5	0.016	0.063	0.25	0.25
Erythromycin	<0.0002	<0.0002	>0.5	>0.5	<0.0002	<0.0002	0.5	0.5

The MIC values of the tested EOs ranged from 0.70 mg/mL (thyme) to 22.66 mg/mL (sage), reflecting varying degrees of antimicrobial efficacy.

For MSSA, rosemary, eucalyptus, lavender, and sage displayed high MIC and MBC values (22.16–22.66 mg/mL), indicating weak antimicrobial and bactericidal activity. Basil EO demonstrated moderate inhibitory potential (MIC = 12.11 mg/mL), but its bactericidal potential was lower (MBC = 24.22 mg/mL). In contrast, thyme EO displayed strong antimicrobial efficacy, with an MIC of 2.81 mg/mL and an MBC of 5.61 mg/mL, highlighting its potent inhibitory and bactericidal properties.

For MRSA, rosemary, eucalyptus, lavender, and sage presented MIC values similar to those observed for MSSA. However, their MBC values were generally higher, indicating limited bactericidal potential. Similarly, basil EO showed the same MIC as for MSSA (12.11 mg/mL), but its MBC exceeded 24.22 mg/mL, reflecting a weaker bactericidal effect. Notably, thyme EO demonstrated exceptional antimicrobial activity, with the lowest MIC and MBC values (0.70 mg/mL), confirming its strong inhibitory and bactericidal effects against MRSA.

For *S. epidermidis*, rosemary, eucalyptus, lavender, sage, and basil exhibited high MIC values (22.16–24.22 mg/mL), indicating weak antimicrobial activity. Additionally, their MBC values exceeded their respective MIC values, further reflecting limited bactericidal potential. Thyme EO, however, demonstrated significantly stronger activity, with an MIC of 2.81 mg/mL and an MBC of 11.22 mg/mL, demonstrating more potent activity than the other EOs. Overall, *S. epidermidis* displayed greater resistance to EOs compared to the other two tested *Staphylococcus* species.

For *C. acnes*, lower MIC and MBC values were observed for rosemary, eucalyptus, and lavender EOs (5.54–5.67 mg/mL), suggesting stronger antimicrobial activity against this strain than against the *Staphylococcus* species. Sage EO displayed the weakest effect, with MIC and MBC values of 22.66 mg/mL. Basil EO showed moderate activity with MIC and MBC values of 12.11 mg/mL, indicating some bactericidal potential. Once again, thyme EO was the most potent, with remarkably low MIC and MBC values (0.70 mg/mL), highlighting its strong inhibitory and bactericidal effects against *C. acnes*.

Overall, thyme EO consistently exhibited the most potent antimicrobial and bactericidal effects across all tested bacterial strains, with notably low MIC and MBC values. In contrast, rosemary, eucalyptus, lavender, and sage demonstrated weaker activity, particularly against *Staphylococcus* species. Basil EO exhibited moderate inhibitory effects but limited bactericidal capacity. *C. acnes* appeared more susceptible to most EOs than *Staphylococcus* strains, with thyme EO emerging as the most effective antimicrobial agent.

Interestingly, antibiotics, particularly chloramphenicol, exhibited lower MIC and MBC values compared to EOs. However, thyme EO presented MIC/MBC values comparable to chloramphenicol against certain strains, namely MRSA and *C. acnes*. In contrast, other oils, such as rosemary and sage, required much higher concentrations to achieve inhibitory or bactericidal effects. Erythromycin proved highly effective against MSSA and *S. epidermidis* but showed no efficacy against MRSA, underscoring antibiotic resistance in this strain. Notably, thyme EO demonstrated strong inhibitory and bactericidal activity against MRSA without any signs of resistance, emphasizing its potential as a natural alternative for addressing antibiotic-resistant bacteria.

Numerous studies have reported the antimicrobial activity of EOs against skin-associated bacteria (26, 28, 29). However, most research has primarily focused on *S. aureus* strains (27, 31, 32), with relatively few studies investigating the effects of EOs on *S. epidermidis* and *C. acnes* (29, 33). Among the tested EOs, thyme consistently exhibited the lowest MIC values for both MSSA and MRSA across various studies, although reported values vary significantly depending on methodological differences and EO composition (26, 28, 32). Consistent with these findings, this study confirmed thyme EO was the most potent antimicrobial agent, further supporting its potential in combating both antibiotic-sensitive and antibiotic-resistant skin pathogens.

Rosemary and eucalyptus EOs are widely recognized for their moderate antimicrobial effects, as reflected by their relatively high MIC/ MBC values compared to thyme EO (28). These findings align with the results obtained in the present study, where both EOs exhibited weaker antibacterial effects than thyme EO.

For *C. acnes*, previous studies have reported highly variable MIC values depending on the EOs and experimental conditions. Juliano et al. (33) obtained an MIC of 4 mg/mL for lavender EO, whereas Esmael et al. (29) reported an MIC of 39 mg/mL for rosemary EO. In the present study, the MIC values for these oils were notably lower, with lavender EO at 5.54 mg/mL and rosemary EO at 5.63 mg/mL, indicating a stronger antimicrobial effect. These variations may stem from differences in EO composition, bacterial strains, or testing methodologies.

The antimicrobial activity of EOs against *S. epidermidis* has been less extensively studied, and available MIC data remain scarce. Esmael et al. (29) reported an MIC value of 78 mg/L for rosemary EO against *S. epidermidis*, a value comparable to its MIC for MSSA but lower than its MIC for *C. acnes*. A similar trend was observed in the present study, reinforcing that *S. epidermidis* may exhibit greater resistance to certain EOs compared to *C. acnes*. These findings highlight the need for further investigations into EO efficacy against *S. epidermidis* to expand the current understanding of their potential therapeutic applications.

### Inhibition curves

**Fig 1:**
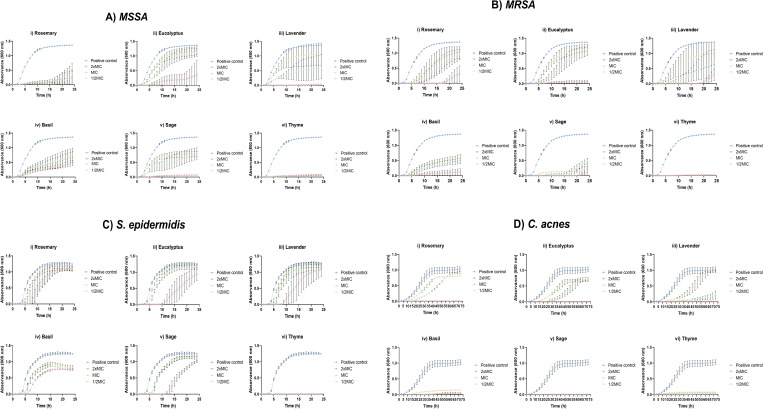
Inhibition curves of EOs agaisnt skin-associated bacteria at 2×MIC, MIC, and ½× MIC: (**A**) MSSA, (**B**) MRSA, (**C**) *S. epidermidis*, and (**D**) *C. acnes*.

The MIC and MBC determinations provided critical insight into the minimum concentration of EO required to inhibit bacterial growth. However, these results from static endpoints did not capture the dynamic behavior of bacteria in response to EOs. To overcome this limitation, growth inhibition curves were generated for each oil at three concentrations (2× MIC, MIC, and ½× MIC) using spectrophotometry (Fig. 1A through D). This approach enabled a more comprehensive assessment of bacterial responses to EOs over time, distinguishing between bacteriostatic and bactericidal effects across the tested concentrations (34).

Analysis of the inhibition curves for MSSA revealed a concentration-dependent effect of all tested EOs, with stronger inhibition observed at higher concentrations. At 2× MIC, the majority of the EOs, particularly lavender, sage, and thyme, completely suppressed MSSA growth, as indicated by flat inhibition. At MIC, significant inhibition was still evident, though some bacterial growth persisted. In contrast, at ½× MIC, inhibition was minimal, with growth patterns resembling those of the positive control, namely for eucalyptus, lavender, and sage EOs. Among the tested EOs, thyme exhibited the strongest antimicrobial activity, maintaining substantial inhibition even at ½× MIC. Eucalyptus and rosemary demonstrated moderate inhibition, particularly at MIC and 2× MIC. While sage and lavender effectively inhibited MSSA at 2× MIC, their inhibitory effect declined at lower concentrations, that is, MIC and ½× MIC. Basil displayed the weakest antibacterial activity, with limited inhibition at both MIC and ½× MIC.

Due to its resistance profile, MRSA exhibited lower susceptibility to EOs compared to MSSA. While significant inhibition was observed at 2× MIC, the effect was less pronounced than in MSSA. At MIC and ½× MIC, bacterial growth was notably higher, with inhibition curves closely resembling those of the positive control, highlighting MRSA’s reduced sensitivity to EOs. Among the tested EOs, thyme demonstrated the strongest antibacterial activity, completely inhibiting MRSA at all tested concentrations. Eucalyptus and rosemary exhibited moderate efficacy, with noticeable growth reduction at 2× MIC and MIC. Sage and lavender demonstrated some inhibitory effects but were less potent against MRSA than MSSA. Basil displayed the weakest inhibition, with minimal impact across all concentrations.

*S. epidermidis* exhibited greater resistance to EOs compared to MSSA and MRSA. Among the tested EOs, only thyme completely inhibited bacterial growth at 2× MIC. At MIC, inhibition was observed but less pronounced, particularly for rosemary, eucalyptus, lavender, and sage EOs. At ½× MIC, bacterial growth was less affected, though thyme EO continued to exert noticeable inhibitory effects. Thyme demonstrated the strongest antimicrobial activity, achieving complete inhibition at 2× MIC and maintaining significant effects even at lower concentrations. Eucalyptus and rosemary were also effective, displaying strong inhibition at 2× MIC and MIC, with moderate inhibition at ½× MIC. In contrast, basil exhibited the weakest antibacterial effect, showing limited inhibition across all tested concentrations.

For *C. acnes*, all EOs exhibited strong inhibition at 2× MIC, indicating bactericidal or highly potent bacteriostatic effects. At MIC, partial inhibition was observed, suggesting a threshold concentration required to effectively control bacterial growth. At ½× MIC, inhibition was significantly reduced, particularly for rosemary and lavender EOs, with growth curves resembling the untreated control, emphasizing the necessity of higher concentrations for sustained antibacterial effects. Among the tested EOs, thyme and sage EOs displayed the highest efficacy, leading to steep declines in bacterial growth at both MIC and 2× MIC. Rosemary, eucalyptus, and lavender exhibited moderate efficacy, delaying bacterial growth at MIC and 2× MIC, though they were less effective than thyme and sage. Basil EO demonstrated strong inhibition, particularly at 2× MIC and MIC, but its effectiveness diminished at ½× MIC.

To complement the graphical data of Fig. 1 and to provide precise quantitative comparisons, inhibition percentages for each EO concentration were calculated at the end of the incubation period (24 h for *Staphylococcus* spp. and 72 h for *C. acnes*). The results are summarized in Table 4.

**TABLE 4:** Inhibition percentages of the EOs at 2× MIC, MIC, and ½× MIC for skin-associated bacteria^*a*^

			EOs					
	Bacteria	Concentrations^*b*^	Rosemary	Eucalyptus	Lavender	Basil	Sage	Thyme
Inhibition percentage (at 24 h)	*MSSA*	2× MIC	93.17 ± 1.14^a^	94.34 ± 2.49^a^	98.53 ± 0.10^c^	60.84 ± 0.73^b^	95.44 ± 0.42^a^	95.55 ± 0.16^a^
		MIC	91.66 ± 2.75^a^	22.15 ± 8.47^d^	68.77 ± 19.6^b^	35.52 ± 9.30^c^	48.46 ± 8.90^b,c^	96.25 ± 0.10^a^
		½× MIC	62.71 ± 1.09^a^	7.90 ± 2.34^e^	54.87 ± 0.00^b^	42.95 ± 8.88^c^	46.03 ± 3.17^c^	95.89 ± 0.00^d^
	*MRSA*	2× MIC	66.48 ± 4.47^a^	95.71 ± 2.84^b^	71.34 ± 6.24^a^	91.28 ± 7.73^b^	93.85 ± 14.5^b^	98.38 ± 0.22^b^
		MIC	36.80 ± 8.21^a,b^	16.07 ± 3.32^c^	30.99 ± 0.94^a,b^	65.19 ± 6.17^b^	64.38 ± 6.24^b^	98.82 ± 0.22^d^
		½× MIC	31.65 ± 10.7^a^	17.61 ± 5.20^b^	12.18 ± 5.61^b^	56.59 ± 7.61^a^	83.88 ± 9.70^c^	98.55 ± 0.26^c^
	*S. epidermidis*	2× MIC	14.26 ± 2.90^a^	42.12 ± 0.00^b^	6.35 ± 0.00^c^	38.88 ± 2.20^b^	17.52 ± 1.59^a^	100.2 ± 0.16^d^
		MIC	15.11 ± 0.23^a^	11.45 ± 0.97^a^	2.73 ± 0.00^b^	39.55 ± 0.24^c^	12.06 ± 0.45^a^	100.4 ± 0.05^d^
		½× MIC	14.83 ± 2.90^a^	9.16 ± 0.00^a^	5.63 ± 2.39^b^	38.06 ± 3.01^c^	9.81 ± 1.14^a^	100.3 ± 0.70^d^
Inhibition percentage (at 72 h)	*C. acnes*	2× MIC	103.4 ± 4.5^a^	101.4 ± 0.7^a,b^	98.29 ± 1.45^b^	95.59 ± 3.35^b^	103.2 ± 3.4^a^	97.88 ± 0.10^b^
		MIC	12.11 ± 1.16^a^	29.09 ± 3.86^b^	78.41 ± 12.3^c^	94.82 ± 0.20^d^	105.2 ± 1.8^e^	92.99 ± 0.34^d^
		½× MIC	20.81 ± 1.16^a^	33.98 ± 3.35^b^	3.716 ± 1.30^c^	85.17 ± 0.34^d^	102.5 ± 1.3^e^	92.15 ± 0.15^d^

^*a*^ Means ± standard deviation within the same row labeled with the same letter do not statistically differ from each other (*P* > 0.05).

^*b*^ The inhibition percentages of all EOs were determined at the minimum inhibitory concentration (MIC), at twice the MIC (2×MIC), and at half the MIC (½×MIC).

For MSSA and MRSA, the inhibition percentages confirmed thyme EO as the most effective oil, demonstrating superior antibacterial activity across all concentrations. The dose-dependent inhibition observed in the graphs was reflected in the table, with 2× MIC resulting in near-complete inhibition (>90% in most oils), while ½× MIC exhibited lower inhibition percentages. Rosemary, eucalyptus, and lavender EOs demonstrated moderate antibacterial activity, with inhibition percentages increasing at higher concentrations, reinforcing their comparative effectiveness against MSSA and MRSA. The percentage data provided a clearer picture of how these EOs compare with thyme.

Data presented in Table 4 also confirmed that *S. epidermidis* was generally more resistant to all tested EOs compared to MSSA and MRSA. This was evident from the consistently lower inhibition percentages at MIC and ½× MIC concentrations. Despite this trend, thyme EO remained the most potent, achieving total inhibition across all tested concentrations, further supporting its superior antimicrobial efficacy.

The inhibition percentages in Table 4 confirmed the strong antibacterial effect of all EOs against *C. acnes*, particularly at higher oil concentrations. Thyme and sage emerged as the most effective, with notable inhibition even at lower concentrations. Interestingly, thyme, basil, and sage maintained relatively high inhibition percentages at ½× MIC, suggesting a lower MIC threshold for *C. acnes* compared to other bacterial species.

While the inhibition curves illustrated bacterial dynamics over time, the inhibition percentages provided a precise snapshot of EO efficacy at specific concentrations, facilitating direct comparisons. *S. epidermidis* exhibited greater resistance to EOs than the other *Staphylococcus* species, whereas *C. acnes* displayed the highest overall inhibition percentages, indicating greater inherent susceptibility. The inhibition data further allowed for a comparative ranking of EO potency: Thyme > Eucalyptus ≈ Lavender > Rosemary > Sage >Basil.

Several studies have assessed the antimicrobial activity of EOs using inhibition curves (5, 29, 35, 36), with MSSA being the most frequently tested bacterium (35, 36). However, these studies did not include the full range of EOs or bacterial strains analyzed in this work, emphasizing the novelty and broader scope of the present study.

For instance, Vázquez-Sánchez et al. (36) investigated the effects of thyme EO at sub-lethal doses against MSSA and found that it slowed bacterial growth. In the present study, thyme EO at sub-MIC also reduced MSSA growth, but the effect was significantly more pronounced. Similarly, Zhang et al. (35) used the same methodology as this study to generate inhibition curves for MSSA exposed to *Cyperus rotundus* EO at 2× MIC, MIC, and ½× MIC. Their findings showed complete inhibition at 2× MIC and MIC, while at ½× MIC, bacterial growth was minimally affected, though the lag phase was extended. Consistently, in the present study, thyme EO nearly eliminated MSSA growth at 2× MIC and MIC, while lavender and sage EOs exhibited similar effects at 2× MIC. Moreover, rosemary EO at ½× MIC followed a growth pattern comparable to that of *Cyperus rotundus* EO in Zhang et al.’s study (35).

Chen et al. (5) examined the inhibition curves of *Litsea cubeba* EO against *C. acnes* using the same methodology as the present research. Their results demonstrated complete bacterial growth inhibition at 2× MIC, while MIC and ½× MIC led to significant but incomplete growth suppression. In agreement with these findings, all tested EOs in this study fully inhibited *C. acnes* at 2× MIC (5). At MIC, eucalyptus and rosemary EOs displayed inhibition patterns comparable to *Litsea cubeba* EO, while at ½× MIC, lavender EO exhibited similar effects (5).

Esmael et al. (29) explored the antimicrobial activity of rosemary EO at 200 mg/L against MSSA, *S. epidermidis*, and *C. acnes* using a different approach—time-kill kinetics. They reported complete bacterial inhibition after 4 h for *C. acnes* and 6 h for MSSA and S. *epidermidis*. In contrast, in the present study, only *C. acnes* was fully inhibited by rosemary, highlighting differences in experimental conditions and bacterial responses.

### Antibiofilm formation activity

Bacterial biofilms are highly resilient microbial communities embedded within a protective extracellular polymeric matrix, making them extremely difficult to eliminate. These biofilms frequently develop on synthetic implants and indwelling medical devices, such as urinary catheters, arthroprostheses, and dental implants, as well as on both living and non-living tissues. Their presence contributes to persistent infections, including endocarditis, otitis media, and chronic wounds (37). The challenge in eradicating biofilms stems from their enhanced resistance to host immune responses and the protective extracellular polymeric substances, which limit antibiotic penetration and facilitate antimicrobial resistance (13). Additionally, the mechanical or chemical disruption of mature biofilms can inadvertently lead to uncontrolled release of bacterial cells and toxins, potentially exacerbating infections (38). Given these challenges, preventing biofilm formation is considered the most effective strategy for mitigating biofilm-associated infections (38).

In this line, EOs have emerged as promising biofilm inhibitors due to their diverse chemical composition, which includes both hydrophobic and hydrophilic molecules. The hydrophobic components of EOs integrate into bacterial cell membranes, disrupting their structural integrity and thereby hindering biofilm formation. Simultaneously, the hydrophilic components can penetrate the exopolysaccharide matrix, further compromising the biofilm structure and enhancing antimicrobial efficacy (12). These dual mechanisms highlight the potential of EOs as effective, natural alternatives for biofilm prevention and control.

**Fig 2:**
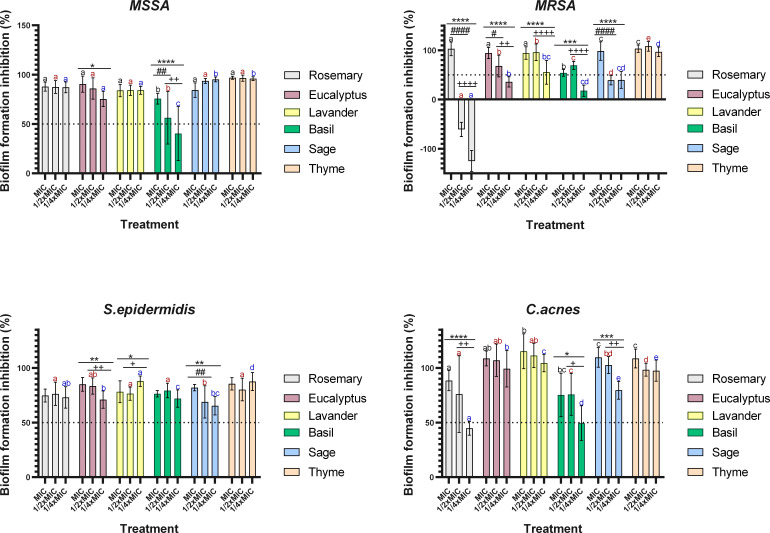
Effect of MIC and sub-MIC concentrations of EOs on biofilm formation in four skin-associated bacteria. Biofilm inhibition is expressed as a percentage. Different letters represent the statistically significant differences between the same concentration of the different EOs (*P* < 0.05), with black letters representing significant differences between MIC for the different oils; red letters indicate significant differences between ½× MIC; and blue letters are the significant differences between ¼× MIC. Symbols represent the significant differences between different concentrations of the same EO. * represents the significant differences between MIC and ¼× MIC (*P < 0.05, **P < 0.01, ***P < 0.001, ****P < 0.0001); # indicates the differences between MIC and ½× MIC (#*P* < 0.05, ##*P* < 0.01, ###*P* < 0.001, ####*P* < 0.0001), and + represents the significant differences between ½× MIC and ¼× MIC (+*P* < 0.05, ++*P* < 0.01, +++*P* < 0.001, ++++*P* < 0.0001).

Therefore, this study evaluated the anti-biofilm activity of all tested EOs at three concentrations—MIC, ½× MIC, and ¼× MIC—against MSSA, MRSA, *S. epidermidis*, and *C. acnes*. The results presented in Fig. 2 show the extent of biofilm inhibition at each concentration. The threshold for considering an EO effective was set at 50% inhibition, with EOs surpassing this value classified as strong biofilm inhibitors, in contrast with moderate or weak inhibition.

For MSSA, all tested EOs demonstrated at least 50% biofilm inhibition at both MIC and ½× MIC. At ¼× MIC, all EOs, except basil, maintained this inhibitory effect, underscoring their strong anti-biofilm potential. Among these, thyme EO exhibited the highest efficacy, achieving nearly 100% biofilm inhibition across all tested concentrations, making it the most potent inhibitor. In contrast, basil EO displayed the weakest anti-biofilm activity, with inhibition showing a clear dose-dependent effect, where inhibition decreased as concentration lowered. Eucalyptus, lavender, and rosemary EOs also exhibited strong biofilm inhibition, though slightly less potent than thyme. These oils followed a dose-response relationship, with higher concentrations leading to greater inhibition. Sage EO, however, demonstrated an inverse trend, where lower concentrations resulted in higher biofilm inhibition, although these differences were not statistically significant (*P* > 0.05). Several studies have documented the antibiofilm activity of EOs against MSSA (26, 28, 36), with thyme EO consistently emerging as one of the most effective (36, 39), which aligns with the findings of this study. Moreover, the dose-dependent inhibitory effects of EOs, as observed for thyme, eucalyptus, lavender, and rosemary, are consistent with previous research (36, 39).

MRSA exhibited the highest variability in response to EOs, with some oils losing effectiveness or even promoting biofilm formation at lower concentrations. At MIC, all tested EOs inhibited at least 50% biofilm formation. However, at ½× MIC, only eucalyptus, lavender, basil, and thyme maintained this inhibition level; at ¼× MIC, only thyme and lavender continued to exhibit at least 50% inhibition. Among the tested EOs, thyme was the most potent, consistently inhibiting over 90% of biofilm formation across all concentrations. Eucalyptus, lavender, and sage EOs also demonstrated strong inhibition at MIC (>90%), but their efficacy declined at ½× MIC, particularly for eucalyptus and sage (*P* < 0.05). At ¼× MIC, the decline in anti-biofilm activity became even more pronounced, with lavender being the only EO maintaining more than 50% inhibition. Basil EO exhibited the weakest anti-biofilm activity, showing only slight inhibition (~50%) at MIC. Interestingly, its inhibition at ½× MIC exceeded that of MIC, though this increase was not statistically significant. At ¼× MIC, biofilm inhibition dropped considerably. In contrast, rosemary EO displayed strong inhibition at MIC (>90%) but lost its inhibitory effect at lower concentrations, even promoting biofilm formation, as indicated by the negative inhibition values observed in the graph.

Brożyna et al. (28) studied the anti-biofilm activity of thyme, basil, rosemary, eucalyptus, and lavender EOs against MRSA. Their findings highlighted thyme, eucalyptus, and rosemary as the most effective oils, while basil was the least active—consistent with the present study. Notably, thyme exhibited the highest anti-biofilm activity across all tested concentrations in both studies, while eucalyptus showed strong inhibition at MIC. However, despite its significant activity at MIC, rosemary EO lacked anti-biofilm effects at sub-MIC concentrations (28). Additionally, Brożyna et al. (28) reported a dose-dependent trend, particularly for thyme and eucalyptus. A similar pattern was also observed in this study for thyme, eucalyptus, and lavender, where biofilm inhibition decreased progressively with lower EO concentrations.

Similar to MSSA, all tested EOs achieved at least 50% biofilm inhibition at MIC and ½× MIC against *S. epidermidis*. Notably, even at ¼× MIC, inhibition remained above 50% for all EOs. However, inhibition percentages at MIC and ½× MIC were generally lower than those observed for MSSA and MRSA, reinforcing the greater resistance of *S. epidermidis* to EOs. Among the tested EOs, thyme exhibited the highest anti-biofilm activity, consistently inhibiting over 80% biofilm formation at all concentrations. Eucalyptus and sage EOs also demonstrated strong inhibition (>80%) at MIC, though their efficacy declined as the concentration decreased. In contrast, rosemary, lavender, and basil EOs displayed moderate inhibition (~70%) across all concentrations, making them the least effective against *S. epidermidis*. Unlike other EOs, these three did not follow a clear dose-response pattern, as their biofilm inhibition did not consistently increase or decrease with concentration.

In this line, some studies have explored the anti-biofilm properties of EOs against *S. epidermidis* (40, 41), though they did not assess as wide a range of EOs as the present study. Abdelhamed et al. (40) specifically studied thyme EO and found it to significantly inhibit *S. epidermidis* biofilm formation, a finding consistent with our study, where thyme EO emerged as the most potent inhibitor of biofilm formation across all tested EOs. Similarly, Karpanen et al. (41) reported that eucalyptus EO exhibited anti-biofilm activity against *S. epidermidis*. Our findings support this, although eucalyptus EO was less effective than thyme EO in biofilm inhibition.

Against *C. acnes*, all tested EOs achieved more than 50% biofilm inhibition at MIC and ½× MIC. However, at ¼× MIC, rosemary and basil were the only oils to fall below this threshold. Once again, thyme EO exhibited the highest potency, consistently achieving near complete (~100%) inhibition across all concentrations, with no significant differences. Eucalyptus, lavender, and sage also demonstrated strong anti-biofilm activity, following a clear dose-response relationship in which inhibition decreased with lower concentrations. In contrast, rosemary and basil EOs exhibited the weakest inhibition, with efficacy dropping below 50% at ¼× MIC.

Overall, *C. acnes* appeared more susceptible to EOs than *Staphylococcus* species, as most oils maintained effectiveness even at lower concentrations. To the best of the authors’ knowledge, no previous studies have specifically evaluated the ability of EOs to prevent *C. acnes* biofilm formation. Existing research primarily focuses on eradicating mature biofilms and did not evaluate the same oils used in this study, underscoring the novelty of the present findings. Oliveira et al. (42) investigated lemon thyme EO’s ability to eradicate mature *C. acnes* biofilms. While their study used a different thyme oil and a distinct anti-biofilm assay, it nonetheless reinforced thyme EO’s strong anti-biofilm potential—consistent with the results of this study.

In summary, the selected EOs effectively prevented biofilm formation across all bacterial species, though their efficacy varied. Thyme EO was the most potent overall, while basil exhibited the weakest activity. Among the bacterial species, *S. epidermidis* demonstrated the highest resistance to EOs, whereas *C. acnes* was the most susceptible, consistently showing higher biofilm inhibition levels.

### Assessment of membrane damage

This study conducted several experiments to evaluate the antibacterial activity of selected EOs against skin-associated bacteria. To further investigate bacterial viability following EO exposure, a flow cytometry assay was performed using the three most promising oils, namely eucalyptus, lavender, and thyme.

**Fig 3:**
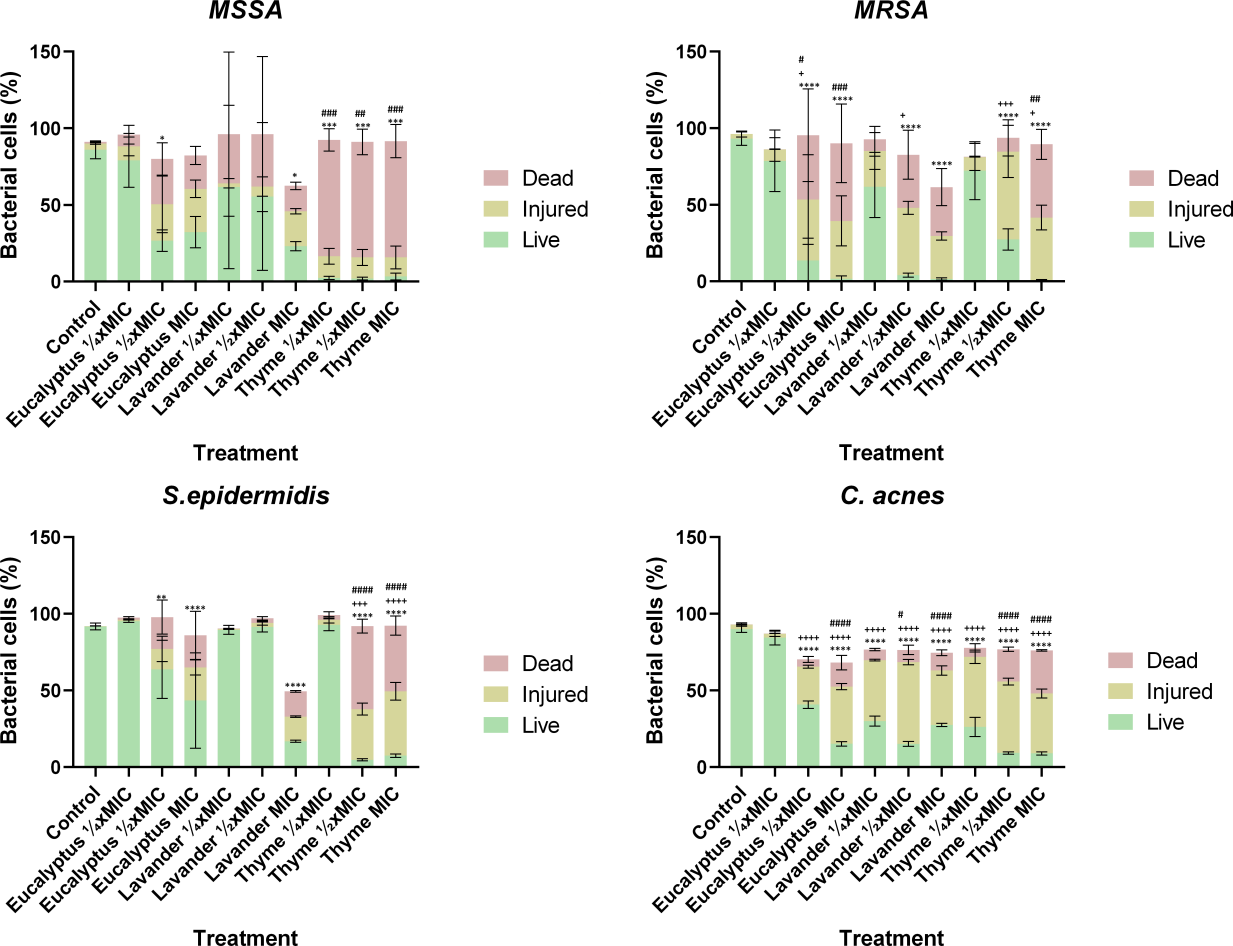
Quantification in percentage of live, injured, and dead bacterial cells after exposure to three concentrations of eucalyptus, lavender, and thyme EOs. A bacterial control without treatment with EOs was also included as a control. Bacterial cells were stained with PI and TO using a live/dead assay, and their viability was quantified by flow cytometry. Statistical significance is indicated as follows: * represent significant differences in live cell percentages between the control and EO-treated groups (*P < 0.05, **P < 0.01, ***P < 0.001, ****P < 0.0001); +represent significant differences in injured cell percentages between the control and EO-treated groups (+*P* < 0.05, ++*P* < 0.01, +++*P* < 0.001, ++++*P* < 0.0001); and # represent significant differences in dead cell percentages between the control and EO-treated groups (#*P* < 0.05, ##*P* < 0.01, ###*P* < 0.001, ####*P* < 0.0001).

Bacterial viability was assessed using a live/dead assay based on PI and TO staining, with flow cytometry quantifying the percentages of live, injured, and dead cells (Fig. 3). This approach also provided insight into EO-induced cell damage, as bacterial cells with intact membranes remain impermeable to PI. In contrast, membrane-compromised cells allow PI uptake, where it binds to DNA and emits fluorescence, serving as a key indicator of membrane integrity loss (43).

Flow cytometry analysis confirmed that thyme EO exhibited the strongest antibacterial activity against MSSA, significantly reducing the percentage of live cells while increasing the proportions of dead and injured cells compared to the control, particularly at MIC and ½× MIC (*P* < 0.05). Lavender EO also demonstrated a notable antibacterial effect, especially at MIC, where a significant increase in dead and injured cells was observed relative to the control (*P* < 0.05). In contrast, eucalyptus EO induced the least cell damage; however, it still led to a significant reduction in live cells at ½× MIC and MIC when compared to the control (*P* < 0.05).

Overall, MRSA showed greater resistance to EOs than MSSA. Thyme EO remained the most effective, causing a significant increase in dead and injured cells at MIC (*P* < 0.05) and a notable rise in injured cells at ½× MIC (*P* < 0.05) compared to the control. Lavender EO also demonstrated strong antibacterial activity, leading to a marked reduction in live cells at MIC and ½× MIC, accompanied by a significant increase in dead and injured cells. Although eucalyptus EO had the weakest effect, it still significantly reduced the percentage of live cells at MIC and ½× MIC compared to the control (*P* < 0.05).

For *S. epidermidis*, thyme EO exhibited the strongest antibacterial activity, significantly increasing the proportion of dead and injured cells compared to the control at MIC and ½× MIC (*P* < 0.05). Lavender and eucalyptus EOs also significantly reduced the percentage of live cells at MIC (*P* < 0.05), with eucalyptus EO additionally causing a notable decrease at ½× MIC (*P* < 0.05). However, none of the tested EOs effectively reduced the percentage of live cells at ¼× MIC. Previous assays indicated that *S. epidermidis* was more resistant to EOs than the other tested bacteria. *S. epidermidis* consistently retained a higher percentage of live cells across all EO treatments, particularly at lower concentrations (¼× MIC and ½× MIC) when compared to MSSA, MRSA, and *C. acnes*. While the MIC led to a significant reduction in live cells, the overall antibacterial effect remained less pronounced than in the other bacterial species.

*C. acnes* consistently exhibited the greatest reduction in live cells across all EO treatments, particularly at MIC and ½× MIC, when compared to the control. Thyme EO demonstrated the strongest antimicrobial effect, significantly decreasing the live cells at all tested concentrations (*P* < 0.05) while increasing the proportions of dead and injured cells. Lavender EO also induced a significant increase in injured cells across all concentrations (*P* < 0.05), though its effect was less pronounced than that of thyme EO. Eucalyptus EO significantly reduced the number of live cells at MIC and ½× MIC compared to the control; however, at ¼× MIC, no visible alterations were observed, as the live cell percentage remained nearly identical to the control.

Consistent with previous findings, *C. acnes* displayed the highest sensitivity to EOs. Flow cytometry further confirmed this trend, revealing a significantly higher proportion of dead and injured cells compared to other bacterial species, underscoring their greater susceptibility to EOs. Additionally, *C. acnes* exhibited notable reductions in live cells and increases in injured cells even at lower EO concentrations, particularly with thyme and lavender EOs—an effect that was not pronounced in the other bacterial species.

These flow cytometry results align with the known mechanisms of action of the main components in the tested EOs. Phenolic compounds like thymol and carvacrol, which are common in thyme EO, are known to target bacterial lipid membranes, increasing permeability and causing leakage of intracellular materials (26, 27). This explains the observed rise in PI uptake and the large number of dead and damaged cells. Likewise, linalool, a primary compound in lavender EO, and 1,8-cineole (eucalyptol), mainly found in eucalyptus EO, are terpenic alcohols and oxides that insert into lipid bilayers, disrupting membrane structure and weakening cellular integrity (26, 44). These physical and chemical effects disturb proton motive force and ion balance, ultimately leading to cell death. Thus, the cytometry findings of membrane damage support the known molecular actions of these key EO components, highlighting their role in the antimicrobial activity of thyme, lavender, and eucalyptus EOs.

The use of flow cytometry to assess the antimicrobial activity of EOs against skin-associated bacteria remains relatively unexplored, with only a limited number of studies employing this technique (38, 45, 46). Moreover, most existing research has primarily focused on *MSSA* (35, 38, 45). To the best of the authors’ knowledge, no previous studies have utilized a live/dead assay combining PI and TO staining, nor have they tested the specific combination of EOs and bacterial species analyzed in this study. These aspects underscore the novelty and significance of the present study. Despite these gaps in the literature, the findings of this study can be correlated with previous research that investigated the antimicrobial effects of other EOs on skin bacteria or employed different viability dyes. For instance, Kang et al. (45) performed a flow cytometry assay on MSSA cells treated with various concentrations of peppermint EO, using SYTO9 and PI staining. Their results demonstrated a dose-dependent increase in the percentage of dead and injured cells following EO exposure. Similarly, in the present study, eucalyptus, lavender, and thyme EOs significantly increased the proportion of dead and injured MSSA cells, particularly at higher concentrations, further supporting the antimicrobial potential of these EOs. 

Likewise, Fu et al. (46) investigated the effects of clove EO on *C. acnes* using PI staining and reported that the oil induced membrane damage, ultimately leading to bacterial cell death. Their study also demonstrated a concentration-dependent increase in the proportion of dead cells. Consistent with these findings, the present study revealed that exposure to eucalyptus, lavender, and thyme EO resulted in a significant reduction in live *C. acnes* cells, with a clear dose-dependent effect. Again, this further reinforces the antimicrobial potential of these EOs and highlights their ability to disrupt bacterial membrane integrity, a key mechanism underlying their anti-biofilm and antibacterial activities.

### Conclusions

EOs are natural bioactive compounds with broad-spectrum antimicrobial activity. They disrupt bacterial membranes, inhibit biofilm formation, and interfere with essential cellular processes. This study demonstrated the antimicrobial potential of rosemary, eucalyptus, lavender, basil, sage, and thyme EOs against four clinically relevant skin-associated bacteria, with thyme EO consistently showing superior efficacy in all assays. It exhibited complete bacterial inhibition in disk diffusion tests, the lowest MIC and MBC values, and the highest bactericidal and antibiofilm activities. Flow cytometry confirmed its strong antibacterial effect, revealing bacterial membrane damage, likely due to its high content of phenolic compounds such as thymol and carvacrol (26, 27, 38).

In contrast, basil EO was the least effective, consistently showing lower bacterial and biofilm inhibition across all tested bacteria. Eucalyptus and lavender EOs displayed strong antimicrobial activity but were less potent than thyme, as indicated by higher MIC and MBC values, lower inhibition percentages, and reduced biofilm disruption. Their effects are primarily attributed to monoterpenes such as 1,8-cineole (eucalyptol) (26) and linalool (44), respectively.

Regarding bacterial susceptibility, *S. epidermidis* exhibited the highest resistance, maintaining a greater proportion of live cells across all treatments, while *C. acnes* was the most susceptible, showing the most significant viability reduction. Notably, EOs were also effective against MRSA, underscoring their potential as alternative treatments for antibiotic-resistant infections.

It is important to note that this study used only one representative reference strain per species. As antimicrobial responses can vary among strains or clinical isolates, this limitation should be addressed in future research, which should include multiple genotypes and clinical isolates to better verify the broader applicability of the observed EO effects.

Additionally, despite these promising findings, EOs face challenges such as high volatility, poor water solubility, and potential risks of skin irritation, including redness, swelling, and burning, which may limit their direct use as topical antimicrobial agents. Furthermore, although the likelihood of resistance development appears lower than with conventional antibiotics, prolonged or sublethal exposure to EOs may still exert selective pressure, potentially promoting tolerance or adaptive microbial responses (47). Therefore, to overcome these limitations and improve their therapeutic potential and practical use as alternatives to antibiotics for skin bacterial infections, future research should focus on targeted delivery systems, such as nanoemulsions, hydrogels, or liposomes (48, 49) that enhance EOs' antimicrobial activity while mitigating their limitations. These delivery systems offer a promising approach to safely and effectively translate EOs into clinical applications for managing skin bacterial infections.
